# First report of *Leishmania* (*Mundinia*) *martiniquensis* in South American territory and confirmation of *Leishbunyavirus* infecting this parasite in a mare

**DOI:** 10.1590/0074-02760220220

**Published:** 2023-05-15

**Authors:** Artur Augusto Velho Mendes, Camila Patrício Braga Filgueira, Luciana de Freitas Campos Miranda, Adilson Benedito de Almeida, Lilian Motta Cantanhêde, Aline Fagundes, Sandro Antônio Pereira, Rodrigo Caldas Menezes, Elisa Cupolillo

**Affiliations:** 1 Fundação Oswaldo Cruz-Fiocruz, Instituto Nacional de Infectologia Evandro Chagas, Laboratório de Pesquisa Clínica em Dermatozoonoses em Animais Domésticos, Rio de Janeiro, RJ, Brasil; 2 Fundação Oswaldo Cruz-Fiocruz, Instituto Oswaldo Cruz, Laboratório de Pesquisa em Leishmanioses, Rio de Janeiro, RJ, Brasil; 3 Fundação Oswaldo Cruz-Fiocruz, Laboratório de Pesquisa Clínica e Vigilância em Leishmanioses, Instituto Nacional de Infectologia Evandro Chagas, Rio de Janeiro, RJ, Brasil

**Keywords:** cutaneous leishmaniasis, horse, Leishmania martiniquensis, Leishbunyavirus, LBV, leishmaniasis epidemiology

## Abstract

**BACKGROUND:**

Epidemiological data related to leishmaniases or *Leishmania* infection in horses are scarce. However, studies carried out in different regions in the world showed equids parasitised by *Leishmania braziliensis*, *L. infantum* and *L. martiniquensis*.

**OBJECTIVES:**

Identify the *Leishmania* species causing cutaneous leishmaniasis in a mare, living in Rio de Janeiro State (Brazil), and search the presence of *Leishmania* viruses in the isolated parasite.

**METHODS:**

Isoenzymes and polymerase chain reaction (PCR) targeting ITSrDNA region followed by sequencing were conducted for typing the isolated parasite. A search for *Leishmania* virus infection was also performed.

**FINDINGS:**

The mare presented skin nodules and ulcers in the left pinna caused by *Leishmania* spp. that was detected by culture and PCR. The parasite was identified as *Leishmania* (*Mundinia*) *martiniquensis*, infected by *Leishbunyavirus* (LBV), representing the first description of this species in South America. The animal travelled to different Brazilian regions, but not to outside the country.

**MAIN CONCLUSIONS:**

The worldwide distribution of *L*. *martiniquensis* and its infection by LBV were confirmed in this study, indicating the autochthonous transmission cycle in Brazil. The clinical profile of the disease in the mare, showing fast spontaneous healing of cutaneous lesions, may indicate that skin lesions related to *L. martiniquensis* infection in horses might be underdiagnosed.

Leishmaniases are, in general, zoonotic infections affecting humans and several domestic and sylvatic animals, causing disease in some of them. Although domestic dogs are the main reservoir of zoonotic leishmaniasis caused by *Leishmania infantum* in urban areas,^1^ several studies have searched for other vertebrate animals, which can play a role in the transmission cycle of *Leishmania* spp.^2^ ^,^ ^3^ ^,^ ^4^ ^,^ ^5^ Among the investigations that look for new possible reservoirs, there were reports on equids showing clinical manifestations of cutaneous leishmaniasis.^6^ ^-^ ^13^ Although *Leishmania* infection in the equid population seems to be sporadic worldwide, the recommendation remains to consider leishmaniases in the differential diagnosis of any papulonodular or ulcerated skin lesion and to perform a test/evaluation of animals transported from endemic to non-endemic areas.^14^

The importance of searching *Leishmania* spp. infections in equids is due to the fact that these animals, as well as dogs and cats, live in close contact with humans. However, epidemiological data related to equine leishmaniasis or *Leishmania* parasites infection in equids are scarce, being most of the time presented only as a clinical case report. Studies carried out in Europe,^8^ ^,^ ^9^ ^,^ ^13^ Asia,^15^ África,^16^ North America^10^ ^,^ ^11^ and South America^2^ ^,^ ^6^ ^,^ ^17^ showed that horses (*Equus caballus*), donkeys (*Equus asinus*), mules (*Equus asinus caballus*) and ponies (*E*. *caballus*) are parasitised by different *Leishmania* species, such as *Leishmania braziliensis*, *L. infantum* and *Leishmania martiniquensis*.

Both *L*. *braziliensis* and *L*. *infantum* are well-known species and most of the time the presence of these species in such area is often associated to several infections in humans and/or domestic animals showing the disease.^2^ ^,^ ^6^ ^,^ ^12^ ^,^ ^18^ ^-^ ^20^ However, some species apparently cause sporadic disease, and it is hard to estimate the prevalence of these species because of the difficulty of detecting infections in animals without clinical signs.

The species *L*. *martiniquensis* was first isolated from human patients with cutaneous leishmaniasis (CL) on the Martinique Island in 1995, described in 2002 but named in 2014.^21^ ^,^ ^22^ More recently, this species was assigned to the subgenus *Leishmania* (*Mundinia*), including other species such as *Leishmania enrietti*, *Leishmania macropodum* and *Leishmania orientalis*.^23^ ^,^ ^24^ ^,^ ^25^ There are still few studies on these species, but one interesting factor is their presence worldwide and their capacity to infect different hosts. Some reports show *L*. *martiniquensis*, previously identified as *Leishmania siamensis,* infecting horses in different parts of the world.^7^ ^,^ ^10^ ^,^ ^11^ Another remarkable feature linked to *L*. (*Mundinia*) species is the description of *L*. *martininquensis* as host of *Leishbunyavirus* (LBV), *Lmar*LBV1, the first virus other than *Leishmania RNA virus* (LRV) found in *Leishmania* species, but found in other trypanosomatid parasites.^26^ Although LRV is considered as a factor that might contribute to the pathogenicity of *Leishmania* spp.,^27^ ^,^ ^28^ it was shown that *Lmar*LBV1 facilitate *L. martiniquensis* infection in vitro.^26^ Several bunyaviruses are causative agents of arthropod-borne diseases of vertebrates and plants.^29^

LBV belongs to the order Bunyavirales, family Leishbunyaviridae, the first non-LRV detected in the genus *Leishmania*. Bunyaviruses were described infecting many trypanosomatid^30^ ^,^ ^31^ ^,^ ^32^ and is large spread in the world, like *L*. (*Mundinia*) species. Structural features of *Lmar*LBV1 are well described by Grybchuk et al.^26^

Here we aim to identify the *Leishmania* species causing CL in a mare living in an area with description of *L. braziliensis* and *L. infantum* transmission. The identification was not achieved by multilocus enzyme electrophoresis, but by single locus sequencing, allowing the first time description of *L*. *martiniquensis* in South America. Considering the information available about *L*. *martiniquensis* bearing LBV,^26^ the presence of this virus was searched and positive result was obtained after polymerase chain reaction (PCR) targeting a LBV fragment.

## MATERIALS AND METHODS

*Case description and biological material collection* - In April 2020, a four-year-old Mangalarga Marchador mare residing in the municipality of Cachoeiras de Macacu, Rio de Janeiro State, Brazil (22º60’36.6 “S 42º83’24.0 “W) was observed presenting multiple cutaneous lesions in the left pinna with a history of difficult healing. The mare was seronegative for equine infectious anaemia virus. The veterinarian in charge of the animal collected material for histopathological examination in a private laboratory, and the report issued highlighted the presence of amastigotes. The veterinarian suspected of CL considering also the animal had a history of traveling in championships to areas endemic for leishmaniases in Rio de Janeiro State, although the mentioned clinical signs were noted only on the farm where the mare lived.

The veterinarians’ team of the Laboratory of Clinical Research on Dermatozoonoses in Domestic Animals (Evandro Chagas National Institute of Infectious Diseases/Fiocruz) was contacted to help in the clinical conduction of this case and collect material for parasitological diagnosis by in vitro parasite isolation and molecular tests. Four skin biopsies were performed using a 4 mm sterile punch on the border of an ulcer observed in the inner surface of left pinna after a local anesthetic block with 2% lidocaine hydrochloride without epinephrine (Hypocaína^®^, Hypofarma, MG, Brazil) and, immediately following, local antisepsis with 2% chlorhexidine digluconate (Riohex^®^, Rioquímica, SP, Brazil), povidone-iodine (Septmax iodopolividona^®^, Farmax, SP, Brazil) and 70% alcohol (Septmax álcool 70%^®^, Farmax, SP, Brazil). Two skin biopsy samples were stored in a sterile plastic tube containing 1 mL of 0.9% Sodium Chloride sterile saline plus antibiotics (1200 U of penicillin and 1000 µg of streptomycin) and antifungal (100 µg of 5’fluorocytocin) stored at 4ºC and sterile saline solution with antimicrobials, maintained refrigerated during transport to the laboratory, for parasitological culture. The other two skin biopsy samples were stored in sterile plastic tubes for PCR and immediately frozen, with any solution. After collection, the samples remained refrigerated during transport to the laboratory.

*Parasite isolation and cultivation* - The diagnostic was also confirmed through parasite isolation in culture. Briefly, skin biopsy samples were cultured in Novy-MacNeal-Nicolle medium plus Schneider’s Drosophila Medium (Sigma-Aldrich, St. Louis, Missouri, USA) supplemented with 10% foetal bovine serum (FBS) and antibiotics penicillin and streptomycin, according to protocol registered in https://dx.doi.org/10.17504/protocols.io.22tggen. The parasite isolated in culture was cryopreserved in liquid nitrogen (N_2_L) and growth of promastigotes cells was performed in sterile bottles for cell culture until the parasites reached the stationary phase of growth. The total culture volume was centrifuged, and the pellet was submitted to three washes in NaCl-EDTA buffer under centrifugation to obtain the parasite mass, which was stored in N_2_L until taxonomic characterisation techniques could be performed. The sample was deposited at the *Leishmania* Collection (CLIOC) from Fiocruz (http://clioc.fiocruz.br) - voucher IOC/L3810.

*Multilocus enzyme electrophoresis (MLEE)* - MLEE was performed on 1% agarose gels supported by GE Healthcare GelBond film (124 x 258 mm), according to previously described procedures,^33^ using four enzymatic systems: 6PGDH (6-phosphogluconate dehydrogenase, EC 1.1.1.43); GPI (glucose phosphate isomerase, EC 5.3.1.9); NH (nucleotidase, EC 3.2.2.1); and PGM (phosphoglucomutase, EC 5.4.2.2). Isoenzyme electrophoresis was performed with the reference strains of *L.* (*V.*) *braziliensis* (MHOM/BR/1975/ M2903) and *L.* (*L.*) *infantum* (MHOM/BR/1974/PP75). Analysis of gel bands was performed qualitatively, by visual comparison of the sample band profiles with the default reference strains.

*Leishmania spp. molecular detection and identification* - Skin biopsy samples and the parasite isolated in culture medium were submitted to DNA extraction and PCR assays for *Leishmania* detection and species identification. DNA was extracted using the *High Pure PCR Template Preparation kit* (Roche, Basel, Switzerland) according to manufacturer’s instructions. PCR targeting *Leishmania* ITS1rDNA was conducted using the same primers and protocols previously described.^34^ ^,^ ^35^ The amplified product was purified using Wizard^®^ SV Gel and PCR Clean-Up System kit (Promega - EUA), following manufacturer instruction. The purified product was sequenced using RPT01A Platform (Fiocruz) and the obtained sequences were analysed using BioEdit.^36^ A nBLAST search (ncbi.gov) was performed to verify the identity of the sequenced sample comparing it to *Leishmania* ITS1rDNA sequences available at the GenBank database (ncbi.gov). The *Leishmania* sequence obtained was deposited at the GenBank under the accession number OP328766. The identity was confirmed by performing phylogenetic analysis including other *Leishmania* species for comparison. The tree was constructed using the Maximum Likelihood method and Kimura 2-parameter,^37^ and 10000 replicates for bootstrap test.^38^ The analysis was performed using MEGA11: Molecular Evolutionary Genetics Analysis version 11.^39^

*LBV detection*: *RNA extraction, cDNA synthesis, PCR, sequencing, and analysis* - To perform the LBV detection, the parasite isolated in culture medium was used. RNA was extracted by TRIzol™ Reagent (Invitrogen, Carlsbad, CA, USA) from 5 mL of culture with 5.10^7^ parasites/mL, as recommended by the manufacturer. The RNA was resuspended in 30 µL of ultrapure water and quantified in NanoDrop (Thermo Fisher Scientific, Wilmington, USA). Two micrograms of total RNA were added to the cDNA synthesis reaction with the High-Capacity cDNA Reverse Transcription Kit (Applied Biosystems, Foster City, CA, USA). For the amplification reaction, 5 µL of cDNA was added to a mix: Platinum™ Taq DNA Polymerase (1.25 U), 10X PCR Buffer (1X), 50 mM MgCl2 (1.5 mM), 10 mM dNTP Mix (0.2 mM each), and primers LBV (0.3 µM each)^26^ and hsp70 for *Leishmania* primers (0.2 µM each)^40^ in an independent amplification reaction. Amplification was performed in 35 cycles (95ºC for 30 s, 56ºC for 45 s, and 72ºC for 45 s), with an initial denaturation and final extension (95ºC for 2 min and 72ºC for 5 min, respectively). The amplification result was visualised on a 2% agarose gel stained with GelRed^®^ Nucleic Acid Gel (Biotium, Fremont, CA, USA). For sequencing, 45 µL of the PCR product was purified with Wizard^®^ SV Gel and PCR Clean-Up System (Promega, Madison, WI, USA) and sequenced on the Fiocruz Sequencing Platform (RPT01A). The LBV sequence was deposited at the GenBank under the accession number OP329212. The identity was confirmed by performing phylogenetic analysis including some LRV and LBV for comparison. The tree was constructed using the Maximum Likelihood method and General Time Reversible model,^41^ and 10000 replicates for bootstrap test.^38^ The analysis was performed using MEGA11: Molecular Evolutionary Genetics Analysis version 11.^39^

## RESULTS

**Fig. 1:**
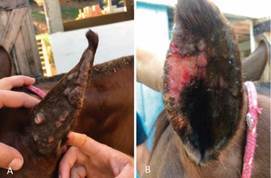
left pinna of a four-year-old Mangalarga Marchador mare. (A) Multifocal to coalescent skin nodules, some ulcerated and oozing in the outer surface; (B) Multifocal skin nodules, some ulcerated and oozing and an ulcer in the inner surface.

In the left pinna were observed multifocal to coalescent skin nodules, some ulcerated and oozing in the outer and inner surface and an ulcer in the inner surface (Fig. 1). These lesions had a history of difficult healing. The mare was in a good general condition without other clinical signs and its routine within the stud farm remained unchanged. Topical treatments with a wound healer and ectoparasiticide based on carbaryl and cypermethrin (Tanicid^®^, Indubras, Minas Gerais, Brazil) and an endectocide based on metrifonate (Neguvon^®^, Bayer, Rio de Janeiro, Brazil) were performed until the complete healing of the lesions for about two months. Approximately twenty other horses living in the same stud farm were clinically evaluated, but no skin lesions were observed on them. In addition, the animal participated in championships in different Brazilian regions, some of which are endemic for canine leishmaniasis caused by *L. infantum*, although it has not travelled outside the country.

Skin fragments taken from the pinna that were collected from the mare and submitted to PCR targeting ITS1rDNA showed no DNA amplification of *Leishmania* spp., despite the amplification for positive controls samples and for the endogenous control targeting SSUrDNA from mammals.

Fragments from cutaneous lesion were seeded in three culture tubes and promastigotes forms of *Leishmania* spp. were visualized on the 22nd day of cultivation in one tube. The other two tubes remained without parasitic growth. Parasitic expansion was carried out and the sample was cryopreserved 24 days after isolation in culture. Only 40 days after parasite isolation in culture, it was possible to have parasites enough to prepare material for MLEE assays.

**Fig. 2:**
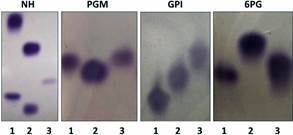
isoenzyme analysis gels of four enzymatic systems: NH (nucleotidase, EC 3.2.2.1); PGM (phosphoglucomutase, EC 5.4.2.2), GPI (glucose phosphate isomerase, EC 5.3.1.9); and 6PGDH (6-phosphogluconate dehydrogenase, EC 1.1.1.43). (1) *Leishmania* (*L*.) *infantum* reference strain (IOC/L579); (2) *L*. (*Viannia*) *braziliensis* reference strain (IOC/L566) and (3) equine sample - IOC/L3810.

In the MLEE, except for PGM enzyme, the observed profile indicated that the sample was different from the reference strains of *L*. *braziliensis* and *L*. *infantum* (Fig. 2). A comparison with a large panel of *Leishmania* strains typed by MLEE at CLIOC was conducted, but the species identification was not achieved.

**Fig. 3:**
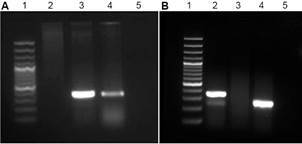
agarose gel electrophoresis showing (A) Polymerase chain reaction (PCR) amplification for the equine *Leishmania* strains IOC/L3810. Lane 1- 100bp; 2- clinical sample* 3- IOC/L3810; 4- positive control; 5- negative control. (B) PCR amplification for *Leishbunyavirus* (LBV). 1- 100bp; 2- LBV fragment^**^; ^**^; 3- negative control; 4- hsp70 fragment (used as a control); 5- blank. ^*^ *Leishmania* PCR was performed using DNA extracted from the skin lesion of the infected mare. ^**^LBV PCR was performed using RNA (transformed in cDNA) extracted from parasite culture.

DNA extracted from the cultivated parasite (IOC/L 3810) was submitted to PCR amplification targeting *Leishmania* ITS1rDNA and a product of approximately 320bp was obtained, as expected (Fig. 3A).

The sequence obtained for the PCR product, sequenced after the amplification of IOC/L3810DNA, was compared with ITS1rDNA *Leishmania* sequences available in the GenBank online database (ncbi.gov), with the BLASTn tool, to detect the *Leishmania* species presenting highest identity with our sample. The results obtained after performing BLASTn search are present on Supplementary data (Table). The results obtained indicated a good identity of IOC/L3810 with *L*. *martiniquensis* and *L*. *siamensis* [Supplementary data (Table)], but the case of *L*. *siamensis* will be discussed latter.

**Fig. 4A-B:**
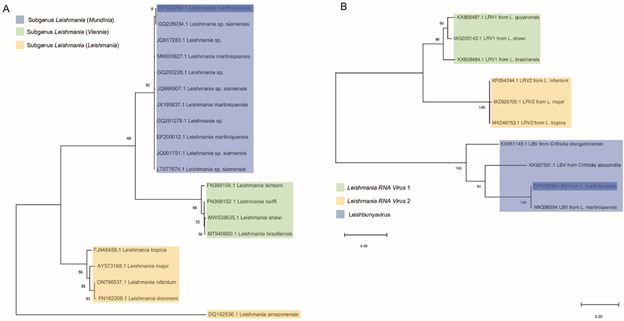
maximum likelihood tree based on (A) ITS1rDNA sequences from different *Leishmania* species and (B) *Leishbunyavirus* (LBV) sequences corresponding to different trypanosomatids. For *Leishmania* sequences the evolutionary history was inferred by using the Maximum Likelihood method and Kimura 2-parameter mode.^37^ Branches corresponding to partitions reproduced in less than 50% bootstrap replicates are collapsed. Initial tree(s) for the heuristic search were obtained by applying the BioNJ method to a matrix of pairwise distances estimated using the Maximum Composite Likelihood (MCL) approach (A). For LBV sequences the evolutionary history was inferred by using the Maximum Likelihood method and General Time Reversible model.^41^ The tree with the highest log likelihood (-40785.87) is shown. The percentage of trees in which the associated taxa clustered together is shown next to the branches. Initial tree(s) for the heuristic search were obtained automatically by applying Neighbor-Join and BioNJ algorithms to a matrix of pairwise distances estimated using the MCL approach, and then selecting the topology with superior log likelihood value. A discrete Gamma distribution was used to model evolutionary rate differences among sites (five categories (+G, parameter = 4.6252). The tree is drawn to scale, with branch lengths measured in the number of substitutions per site (B). For both tree the bootstrap was inferred from 10000 replicates. All the sequences analysed are presented using their corresponding GenBank accession number.

Phylogenetic analysis adding other *Leishmania* species for comparison was conducted. Twenty sequences, including the obtained in this study, were used in the analysis, representing species from the subgenus *L*. (*Mundinia*), *L*. (*Viannia*), and *L*. (*Leishmania*). The *Leishmania* strain IOC/L3810 (Accession number OP329212) clustered together with all ten *L*. (*Mundinia*) sequences analysed, identified as *L. martiniquensis*, *Leishmania* sp. *siamensis*, and *Leishmania* sp. (Fig. 4A-B).

Considering the information available about the presence of LBV in *L*. *martiniquensis*,^26^ was searched the presence of this virus and positive result was obtained after PCR targeting a LBV fragment (Fig. 3B). The LVB sequencing result (288 bp) was submitted to BLASTn search and are presented on Supplementary data (Table) (gray line), showing 98.61% identity with *L. martiniquensis leishbunyavirus* 1 (MK356554.1). The sequence from the virus detected infecting the strain IOC/L3810 clustered with other LBV from *Crithidia* species, but with higher similarity with the LBV sequence from *L*. *martiniquensis* public available (Fig. 4A-B).

## DISCUSSION

The species *L. martiniquensis* was first described causing diffuse nodular CL in an HIV-infected patient in the Martinique Island,^22^ but one year after this parasite was also associated to visceral leishmaniasis in an HIV patient in the Caribbean.^42^ This species was already found infecting humans in Thailand and Myanmar,^43^ but also infecting cow in Switzerland^44^ and horses in Germany, Switzerland and Florida.^9^ ^,^ ^10^ ^,^ ^11^ The name *L. siamensis* was employed in some of these studies, but this name is now representing a synonymy of *L*. *martiniquensis.* ^25^ This species was described as belonging to the subgenus *Leishmania*,^22^ but later was assigned to the subgenus *L.* (*Mundinia*), which includes other species.^23^

South America comprehends a large fraction of global biodiversity, representing one of the most species-diverse regions on Earth.^45^ This rich environment contributes to the maintenance, dispersion, and diversity of *Leishmania* species.^46^ ^,^ ^47^ Except for *Sauroleishmania*, members of all *Leishmania* subgenera were observed in this region. So far, *L*. *enriettii* is the only species from the *Mundinia* subgenus described in a South American country, with some reports of this species in Brazilian areas.^23^ ^,^ ^48^ Species classified in the subgenus *Mundinia* are closely related among them, but distinct from species from other subgenera. This subgenus includes species widely dispersed geographically, covering all continents, excluding only Antarctica:^23^ *L*. *enriettii*,^49^ *L*. *martiniquensis*,^22^ ^,^ ^39^ *L*. *macropodum*,^50^ *L*. *orientalis*,^24^ and *L*. sp. from Ghana.^51^ Among these four species, *L*. *martiniquensis* is the most spread species, but for the first time is being described in a South American region, in southeast Brazil.

Equine species may also have become part of the transmission chain as a potential reservoir of *Leishmania* species in some regions.^14^ ^,^ ^52^ Considering the published studies, the main clinical manifestations of equine leishmaniasis were limited to the skin and included ulcers, nodules, crusts, papules, areas of alopecia, presence of exudate and pruritus.^14^ Most lesions were described as multiple,^6^ ^,^ ^7^ ^,^ ^8^ ^,^ ^9^ ^,^ ^10^ ^,^ ^11^ although single lesions were also observed in some studies,^9^ ^,^ ^12^ ^,^ ^13^ distributed over different parts of the body.^53^ The importance of research on leishmaniasis in equids lies in the fact that these domestic animals, such as dogs and cats, are in close contact with humans, which may be through their use as a means of transport or for work, companion for leisure activities, breeding, animal assisted therapy, production of meat as food source and sports.^54^

For sports competitions, the horses may be frequently transported to other regions of the same country or abroad, increasing the risks of spreading infectious diseases^55^ such as leishmaniases.^14^ Therefore, the mare of this study may have been infected due to travel for sports competition to the region of Itaipuaçu, in the municipality of Maricá, State of Rio de Janeiro, Brazil, that is endemic for leishmaniases.^56^ ^,^ ^57^ Unfortunately, epidemiological data on equine leishmaniasis are scarce and mostly recorded as clinical case reports.^13^ ^,^ ^14^

The skin lesions and their location associated to *L. martiniquensis* infection are non-specific and similar to that observed in other cases of leishmaniases in horses caused by *L. martiniquensis*,^9^ ^,^ ^10^ ^,^ ^11^ *L. braziliensis*,^6^ and *L. infantum* ^7^ ^,^ ^8^ ^,^ ^13^ as other cutaneous diseases such as neoplasms, mycoses, habronemiasis or hypersensitivity reactions.^8^ Therefore, it is very important to perform accurate laboratory tests that confirms the infection and allow the identification of the involved species of *Leishmania* spp. such as PCR and parasitological culture that were performed in this study. The importance of *Leishmania* species identification is reinforced considering that the municipality of Cachoeiras de Macacu is endemic for *L. braziliensis* and had the first non-autochthonous case of canine visceral leishmaniasis caused by *L. infantum* in 2011.^58^

An interesting factor associated to *L*. *martiniquensis* is its possible infection by a RNA virus named LBV, LmarLBV1, detected in the reference strain from Martinique (MHOM/MQ/92/MAR1), but viruses were not found in strains representing other *L.* (*Mundinia*) species evaluated.^26^ As far as we know, no searches were conducted to evaluate the presence of LBV in other *L*. *martiniquensis* strains available, but herein we observed that the *L*. *martiniquensis* isolated from the Brazilian horse was infected by LBV. The impact of infection of *L*. *martiniquensis* by LBV was little explored, but it was demonstrated that the presence of this virus is somewhat advantageous for *Leishmania*.^26^ Another interesting aspect of *L*. *martiniquensis* is its association with asymptomatic infection without clinical signs and also with cutaneous and visceral leishmaniases in humans, but this spectrum of infection is not essentially associated with HIV.^59^ Although we did not observe a severe disease in the horse infected by *L*. *martiniquensis* in this study, we cannot exclude the relevance of LBV for leishmaniases pathogenesis as already demonstrated for *Leishmania* species infected by LRV.^27^

Studies looking for sand flies in the region where we found the infected mare reported the presence of species belonging to *Lutzomyia intermedia* complex.^60^ In the municipality of Cachoeiras de Macacu, Rio de Janeiro, the presence of *Migonemyia migonei* that is vector of American Tegumentary Leishmaniasis (ATL) has also been detected.^61^ These findings are important because, although the mare has a history of traveling, it spends most of the time in the stud farm, an area suitable for transmission of some *Leishmania* spp.

Although phlebotomine sand flies considered vectors of most *Leishmania* species, vectors of species from subgenus *Mundinia* are still unknown. The biting midges of the genus *Culicoides* (Diptera: Ceratopogonidae) are possible vectors since *C. sonorensis* was experimentally infected by all species of the subgenus *Mundinia* and was able to transmit *L. martiniquensis*, *L. orientalis* and *L*. sp. from Ghana to mice through their bites, which was not observed for phlebotomine sand flies.^62^

*In conclusion* - The worldwide distribution of *L*. *martiniquensis* and its infection by LBV were confirmed in this study, indicating the autochthonous transmission cycle in Brazil. The clinical profile of the disease in the horse, showing fast spontaneous healing of cutaneous lesions, may indicate that skin lesions related to *L. martiniquensis* infection in horses might be underdiagnosed.
