# Supplementary material for: First report of Leishmania (Mundinia) martiniquensis in South American territory and confirmation of Leishbunyavirus infecting this parasite in a mare
Source: Mem Inst Oswaldo Cruz. 2023 May 15;118:e220220. doi: 10.1590/0074-02760220220 (PMC10194060; doi:10.1590/0074-02760220220)
Supplement: Supplementary file 1 [file 1678-8060-mioc-118-e220220-s.pdf]

TABLE

Comparison of the sequence obtained for the polymerase chain reaction (PCR) product of the skin samples of cutaneous lesions of the left pinna in a mare and of *Leishbunyavirus* (LBV) with ITS1rDNA *Leishmania* and LBV sequences available in the GenBank online database using the BLASTn tool

Analysed strain	Target	Description	Scientific name	Max score	Total score	Query cover	E value	Identity	Accession
IOC/L3810	ITS1rDNA	*Leishmania martiniquensis* isolate PCM4 18S ribosomal RNA gene, partial sequence; internal transcribed spacer 1, complete sequence; and 5.8S ribosomal RNA gene, partial sequence	*Leishmania martiniquensis*	588	588	99%	1,00E-163	97.95%	JX195637.1
		Leishmania sp. H1 18S ribosomal RNA gene, partial sequence; internal transcribed spacer 1, complete sequence; and 5.8S ribosomal RNA gene, partial sequence	*Leishmania* sp. H1	582	582	99%	6,00E-162	97.66%	GQ281278.1
		L. martiniquensis internal transcribed spacer 1 and 5.8S ribosomal RNA gene, partial sequence	*Leishmania martiniquensis*	573	573	99%	3,00E-159	97.09%	EF200012.1
		Leishmania sp. CR-2012 strain Ec11010 internal transcribed spacer 1, partial sequence	*Leishmania* sp. *'siamensis'*	542	542	91%	9,00E-150	97.78%	JQ617283.1
		Leishmania sp. *siamensis* clone LEBM-So 18S ribosomal RNA gene, partial sequence; internal transcribed spacer 1, complete sequence; and 5.8S ribosomal RNA gene, partial sequence	*Leishmania* sp. *'siamensis'*	525	525	89%	9,00E-145	97.72%	JQ001751.1
		L. martiniquensis isolate ASKBE3-1 small subunit ribosomal *RNA* gene, partial sequence; internal transcribed spacer 1, complete sequence; and 5.8S ribosomal RNA gene, partial sequence	*Leishmania martiniquensis*	523	523	89%	3,00E-144	97.72%	MK603827.1
		Leishmania sp. *siamensis* clone LE-San1 18S ribosomal RNA gene, partial sequence; internal transcribed spacer 1, complete sequence; and 5.8S ribosomal RNA gene, partial sequence	*Leishmania* sp. *'siamensis'*	520	520	89%	4,00E-143	97.39%	JQ866907.1
		Leishmania sp. LECU1 internal transcribed spacer 1 and 5.8S ribosomal RNA gene, partial sequence	*Leishmania* sp. LECU1	520	520	89%	4,00E-143	97.39%	GQ293226.1
		Leishmania sp. *siamensis* internal transcribed spacer 1 and 5.8S ribosomal RNA gene, partial sequence	*Leishmania* sp. *'siamensis'*	520	520	89%	4,00E-143	97.40%	GQ226034.1
		Leishmania sp. *'siamensis'* genomic DNA sequence contains 18S rRNA gene, ITS1, 5.8S rRNA gene	*Leishmania* sp. *'siamensis'*	518	518	88%	2,00E-142	97.69%	LT577674.1
IOC/L3810	LBV	*L. martiniquensis leishbunyavirus* 1 strain OSU10 segment L RNA-dependent RNA polymerase gene, complete	*Leishmania martiniquensis* leishbunyavirus 1	510	510	100%	3e-140	98.61%	MK356554.1

CC BY
